# Multiparametric CMR in Myocarditis: A Comprehensive Review of Diagnostic Advances, Prognostic Value, and the Challenge of Genetic Mimics

**DOI:** 10.3390/biomedicines14030588

**Published:** 2026-03-05

**Authors:** Wissam Alam, Houssem Hamrouni, Ivelina Choneva, Cyrus Moini

**Affiliations:** 1 Cardiology Department, Groupe Hospitalier Sud Ile de France, 77000 Melun, France; cyrus.moini@ghsif.fr; 2 Radiology Department, Groupe Hospitalier Sud Ile de France, 77000 Melun, France; houssem.hamrouni@ghsif.fr (H.H.); ivelina.choneva@ghsif.fr (I.C.)

**Keywords:** myocarditis, cardiac MRI, Lake Louise criteria, late gadolinium enhancement, T1 mapping, T2 mapping, inflammatory cardiomyopathy, parametric mapping, future perspectives

## Abstract

Cardiac magnetic resonance (CMR) has revolutionized the diagnosis and risk stratification of myocarditis. Beyond its capacity in detecting functional abnormalities, CMR is now capable of tissue characterization with high sensitivity and specificity, allowing for the identification of myocardial edema, hyperemia, and necrosis/fibrosis. The introduction of the Lake Louise Criteria (LLC), updated in 2018 with the integration of parametric mapping techniques, has significantly improved diagnostic sensitivity and specificity. This review details the definition, stages, and etiologies of myocarditis, exploring the diagnostic journey from early techniques to the modern multiparametric approach. It underscores the prognostic value of CMR findings and highlights challenging scenarios such as genetic cardiomyopathies that mimic myocarditis. It then discusses CMR patterns in specific conditions like cardiac sarcoidosis, amyloidosis, and immune checkpoint inhibitor-induced myocarditis. Finally, future perspectives for CMR in myocarditis are addressed.

## 1. Introduction

Myocarditis is defined as an inflammatory disease of the heart muscle diagnosed by histological, immunological, and immunohistochemical criteria, known as the “Dallas Criteria” [1]. Clinically, it exhibits a broad range of presentations, from subclinical forms to fulminant heart failure, cardiogenic shock, and sudden cardiac death. The pathophysiological process involves myocardial cell inflammation, injury, and necrosis. Myocarditis may have various etiologies (Table 1), both infectious and non-infectious, and can present in acute, subacute, or chronic phases. Unfortunately, it has many mimics due to the lack of specific markers. Endomyocardial biopsy (EMB) remains the gold standard for diagnosis [2]; however, because of its invasive nature, it is seldom used outside exceptional circumstances. In addition, EMB is most frequently performed in the right cardiac chambers and results in numerous false-negative studies. Cardiac MRI has emerged as a strong alternative and is recommended as the first-line exam in suspected myocarditis according to the 2025 European and 2024 American guidelines. MRI can confirm a definitive diagnosis when compatible clinical criteria are present [3,4]. In this review article, we discuss the various MRI criteria for myocarditis at each disease stage, including both the old and new Lake Louise criteria. We highlight the limitations of this modality, especially regarding certain genetic disorders that can mimic myocarditis. We also focus on specific etiologies and their characteristic MRI findings and conclude by exploring future directions and perspectives.

## 2. Materials and Methods

### 2.1. Study Design and Search Strategy

This article is a comprehensive narrative review focused on the evolving role of CMR imaging in the diagnosis and management of myocarditis. A comprehensive literature search was performed using the PubMed and Google Scholar databases to identify the relevant literature.

The search strategy employed the following specific keywords and their combinations: “myocarditis”, “cardiac MRI”, “Lake Louise Criteria”, “cardiac magnetic resonance”, “myocardial inflammation”, “T1 mapping”, “T2 mapping”, “inflammatory cardiomyopathy”, “parametric mapping”, “future perspectives”, and “late gadolinium enhancement”.

### 2.2. Inclusion and Exclusion Criteria

To determine the relevance of the articles for this review, strict inclusion and exclusion criteria were applied.

Inclusion criteria:

○ Original research articles, comprehensive reviews, meta-analyses, and official clinical guidelines.
○ Studies specifically addressing CMR diagnostic criteria (including the 2009 and 2018 Lake Louise Criteria), multiparametric imaging techniques, differential diagnosis (including genetic mimics), and prognostic implications of CMR findings.
○ Articles exploring future perspectives in CMR, such as quantitative imaging advancements and artificial intelligence.
○ Articles published up to February 2026.

Exclusion criteria:

○ Articles where the primary focus was not on myocarditis or cardiac imaging.
○ Single-case reports (unless detailing highly novel or rare specific patterns, such as early immune checkpoint inhibitor toxicities).
○ Abstracts, conference presentations, or opinion pieces without available full-text peer-reviewed data.
○ Studies with insufficient data regarding CMR parameters.

### 2.3. Article Selection and Language Processing

Articles were initially considered without language restrictions. A total of 4 articles were originally in French. These non-English articles were comprehensively reviewed and translated by the bilingual authors (W.A. and H.H.) to ensure accurate data extraction and inclusion. However, all of the final selected articles were in English.

### 2.4. Study Selection Pathway

The initial database search yielded a total of 1245 articles. After the removal of duplicates (182 articles), 1063 unique records were screened by title and abstract. During this phase, 895 articles were excluded as they did not meet the inclusion criteria or fell under the exclusion criteria. The remaining 168 full-text articles were assessed for eligibility. Finally, 71 articles were deemed highly relevant and were included in this comprehensive review.

**Figure 1:**
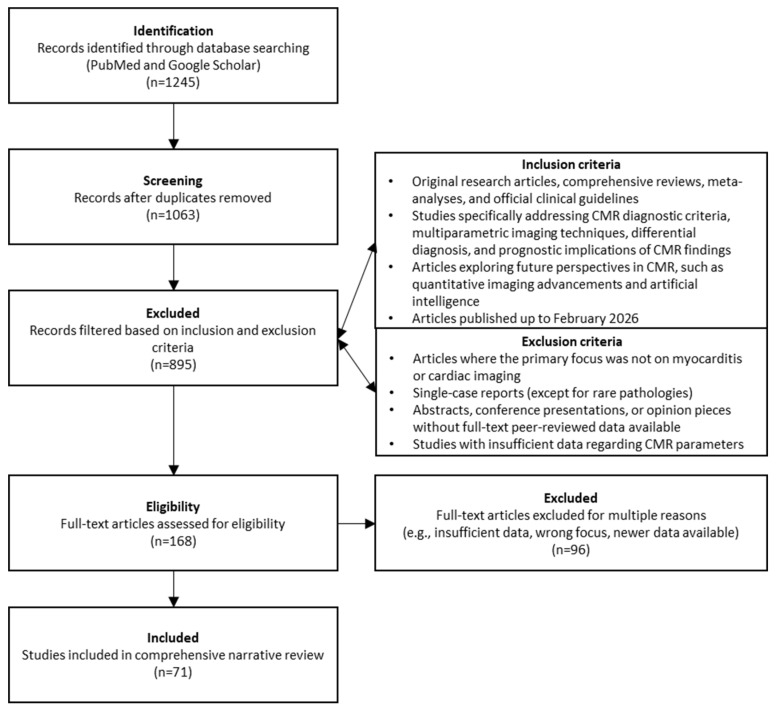
Flow chart detailing literature search and selection pathway used in this review.

The detailed literature search and selection pathway is illustrated in the flow chart below (Figure 1).

## 3. Stages of Myocarditis

**Figure 2:**
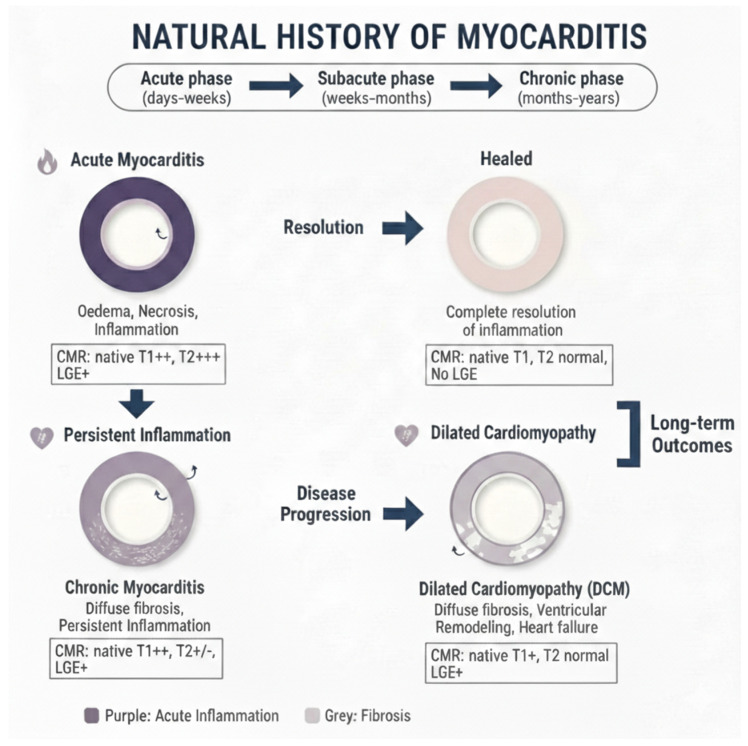
Natural course of myocarditis as seen by cardiovascular magnetic resonance using quantitative techniques and late gadolinium enhancement.

The evolution of myocarditis depends on pathogen-specific factors, the nature of the initial myocardial insult, and the patient’s unique genetic and immunological profile and can evolve in several stages (Figure 2). Some patients will have a moderate form of myocarditis that rapidly resolves with or without sequela, and others will have a severe, fulminant form requiring circulatory support, while others will end up having a chronic form of myocarditis.

**Figure 3:**
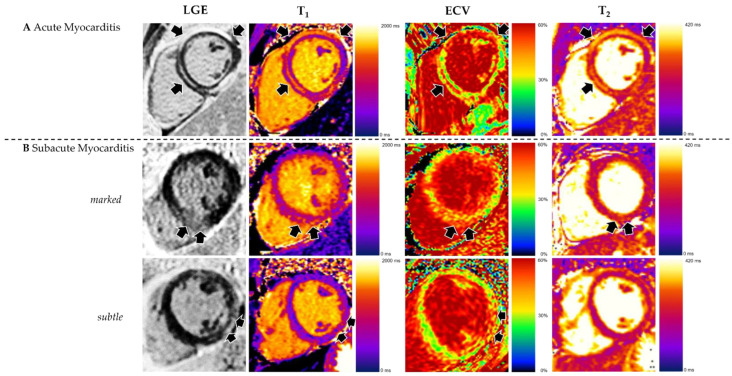
Appearance of acute and subacute myocarditis in CMR. (**A**) Changes are more pronounced in acute myocarditis, as demonstrated by high prevalence and extent of late gadolinium enhancement (LGE) and elevated T2. (**B**) Subacute myocarditis can show increased LGE as well as elevated T1, ECV, and T2, but in many cases, changes are subtle. Reproduced from: Brendel, J.M., et al. (2022) [6]. Licensed under CC BY 4.0. (https://creativecommons.org/licenses/by/4.0/ accessed on 15 December 2025).

**Figure 4:**
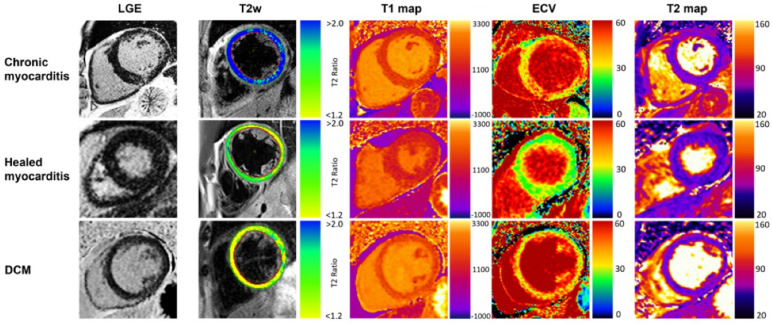
Typical CMR findings of chronic healed myocarditis and DCM. T2w and T2 mapping show marked edema in chronic myocarditis contrary to healed myocarditis and DCM. Increased T1 relaxation times and ECV were present in all groups, prominently depicted in the chronic myocarditis and DCM examples. Reproduced from: Krumm, P., et al. (2022) [10]. Licensed under CC BY 4.0. (https://creativecommons.org/licenses/by/4.0/, accessed on 15 December 2025).

- Acute Myocarditis: This initial stage, typically lasting less than a month, is characterized by active myocardial inflammation. Pathologically, this involves myocyte injury either directly by the inciting agent (e.g., virus) or indirectly by the triggered immune response, leading to intracellular edema, hyperemia, and early necrosis [5]. Clinically, patients may present with chest pain, heart failure symptoms, or arrhythmias. CMR at this stage typically shows myocardial edema, early gadolinium enhancement (EGE), and late gadolinium enhancement (LGE) [6] (Figure 3). From a biochemical standpoint, acute myocarditis is typically accompanied by elevation of myocardial necrosis markers, predominantly cardiac troponins, which reflect the extent of myocyte injury but only moderately correlate with CMR tissue damage [7,8,9]. In a cohort of patients with acute myocarditis, peak cardiac troponin T showed a linear association with total LGE volume (R^2^ ≈ 0.57, *p* < 0.001), whereas no significant correlation was found with the T2 signal intensity ratio, underscoring that LGE is a better marker of irreversible injury than edema. Conversely, another study reported that the proportion of LGE-positive myocardium did not correlate with the amount of necrosis estimated from troponin T release, highlighting the imperfect relationship between focal scar on CMR and global biochemical injury. Overall, troponin elevation supports the diagnosis in the acute phase, but CMR—through LGE and mapping—more accurately characterizes the pattern and extent of myocardial involvement.
- Subacute Myocarditis: This is a transitional phase (duration of symptoms > 4 weeks to <3 months) where the acute inflammatory response begins to subside. There may be a mix of persistent edema and the early establishment of repair processes, including the beginning of fibrotic scar formation. In the subacute phase, serum troponin and inflammatory markers usually decline or normalize, while CMR often still demonstrates residual abnormalities, indicating a temporal dissociation between enzymes and imaging [6,9]. Follow-up CMR studies in patients approximately 3 months after an acute episode have shown persistent LGE or increased native T1/ECV in a significant proportion of cases despite normalization of cardiac enzymes and CRP, reflecting ongoing structural remodeling rather than active myocyte necrosis. In one series, CMR frequently revealed persistent disease activity at 3-month follow-up even though cardiac enzymes and inflammatory parameters had returned to normal, emphasizing that multiparametric CMR is more sensitive than circulating biomarkers for detecting subacute myocardial injury.
- Chronic Myocarditis: With persistent inflammation and symptoms lasting more than 3 months, this stage can lead to a chronic inflammatory cardiomyopathy [3]. The ongoing inflammation, usually driven by autoimmune mechanisms, results in progressive myocardial damage, diffuse interstitial fibrosis, and systolic dysfunction. CMR may show persistent or fluctuating edema on T2-weighted sequences alongside LGE [10] (Figure 4). In chronic myocarditis and inflammatory cardiomyopathy, classical myocardial enzymes are often normal or only mildly elevated, and their correlation with CMR findings becomes weak. Patients may exhibit persistent non-ischemic LGE and diffusely increased native T1 and ECV—reflecting fibrosis and low-grade inflammation—despite normal troponin and CK levels, indicating that tissue characterization by CMR is more informative than enzymes for assessing ongoing disease activity in this stage. Conversely, systemic inflammatory markers such as CRP or ESR can be variably elevated and show only a loose relationship with mapping or LGE burden, so current evidence supports using CMR (and, when appropriate, PET) as the reference non-invasive tool to monitor chronic inflammation and remodeling, while serum biomarkers play a complementary role [9,10,11,12].
- Resolved Myocarditis: In many cases, particularly viral, the inflammation resolves completely without significant sequelae. Follow-up CMR may show complete normalization of T1, T2, and increased extracellular volume (ECV) values and the absence of new LGE.
- Fibrosis and Dilated Cardiomyopathy (DCM): A long-term consequence in a subset of patients is the development of myocardial fibrosis, either focal (replacement fibrosis) or diffuse (interstitial). This fibrotic remodeling can lead to a DCM with impaired left ventricular (LV) systolic function, representing the end-stage of the inflammatory process [13]. LGE, particularly mid-wall or subepicardial, is a hallmark of this stage.

## 4. The Role of CMR in the Diagnosis of Myocarditis

Cardiac magnetic resonance assessment for myocarditis proceeds through a logical sequence of imaging steps, each targeting specific myocardial changes. The process begins with cine imaging (steady-state free precession sequences in multiple planes) to measure ventricular function—ejection fraction, end-diastolic volume, stroke volume, and regional wall motion—establishing whether systolic impairment or focal hypokinesis accompanies the inflammatory process. Next comes T2-weighted imaging (typically short tau inversion recovery, STIR), which highlights myocardial edema as areas of high signal intensity relative to skeletal muscle; this reflects active water accumulation from inflammation and capillary leak, most prominent in acute disease. Native T1 mapping and extracellular volume (ECV) quantification follow, detecting diffuse fibrosis, myocellular damage, or protein accumulation through prolonged relaxation times; these quantitative measures excel at identifying global injury missed by focal techniques. Early gadolinium enhancement (EGE), acquired 2–5 min post-contrast, reveals myocardial hyperemia by comparing signal intensity to normal myocardium or skeletal muscle, though its use has declined due to inconsistent performance. Finally, late gadolinium enhancement (LGE) imaging at 10–20 min post-contrast identifies irreversible injury—appearing as non-ischemic (subepicardial/mid-wall) hyperenhancement—serving as both diagnostic and prognostic cornerstones across disease phases. When ≥2 abnormal findings align with clinical suspicion, the Updated Lake Louise Criteria confirm myocarditis with high specificity.

The initial use of CMR in myocarditis was primarily functional, utilizing cine imaging to assess ventricular volumes, ejection fraction, and regional wall motion abnormalities. While echocardiography remains the first-line tool for functional assessment, CMR’s superiority in tissue characterization quickly became apparent. The development of T2-weighted imaging to detect edema and the introduction of gadolinium-based contrast agents to identify areas of hyperemia and necrosis/fibrosis laid the groundwork for a tissue-based diagnosis [14].

The need for a standardized diagnostic approach led to the establishment of the original LLC in 2009 [15]. These criteria required at least 2 of the following 3 findings for a positive CMR diagnosis:

- Regional or global myocardial edema on T2-weighted images (e.g., T2 signal intensity ratio ≥ 2.0).
- Global myocardial hyperemia and capillary leak on EGE.
- At least one focal non-ischemic lesion on LGE, typically in a subepicardial or mid-myocardial distribution.

While specific, the original criteria had limitations, including the subjective nature of EGE and T2-weighted imaging, susceptibility to artifacts, and reduced sensitivity in diffuse disease.

The 2018 Updated Lake Louise Criteria integrated quantitative parametric mapping techniques, marking a significant advancement [9]. The new criteria recommend a “2 out of 2” approach, requiring at least one T1-based criterion AND one T2-based criterion:

- T1-based criterion (indicating myocardial injury, necrosis, or fibrosis):
  ○ Elevated native T1 relaxation times.
  ○ Presence of non-ischemic LGE.
  ○ Increased ECV.
- T2-based criterion (indicating myocardial edema):
  ○ Elevated native T2 relaxation times.
  ○ Regional or global increase in T2 signal intensity on T2-weighted images.

**Figure 5:**
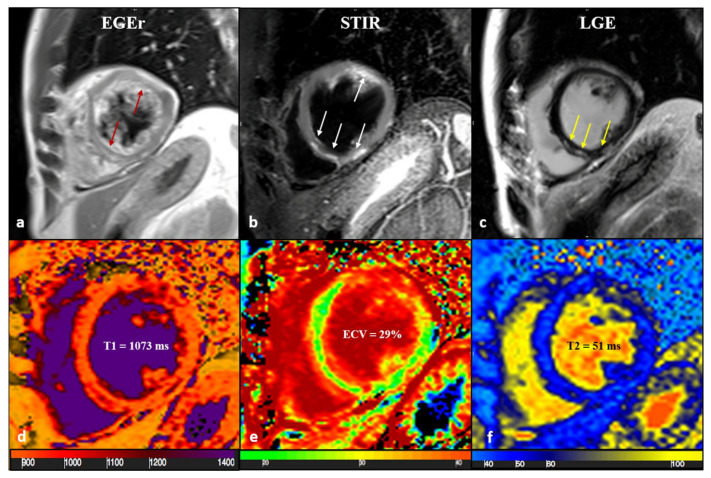
A 43-year-old male patient with acute chest pain and T-Troponine release (0.05 mcg/L). The EGE ratio (**a**) was 5.4, with hyperemia of the anterior and infero-septal segments on the mid-ventricular plane (**a**, red arrows). Edema (**b**) with a patchy distribution on the antero-lateral and infero-septal segments (**b**, white arrows) was confirmed by a T2 ratio of 2.4 on the mid-ventricular plane. A non-ischemic LGE (**c**) was found in the inferior and infero-septal segments (**c**, yellow arrows) with a subepicardial distribution. Native T1 (**d**, mean value of 1073 ms), ECV (**e**, mean value of 29%), and T2 mapping (**f**, mean value of 51 ms) were all increased, confirming the positivity of both old and new LLC. EGE—early gadolinium enhancement, STIR—short tau inversion recovery, LGE—late gadolinium enhancement, ECV—extracellular volume fraction, and LLC—Lake Louise Criteria. Reproduced from: Cundari, G., et al. (2021). [17]. Licensed under CC BY 4.0 (https://creativecommons.org/licenses/by/4.0/ accessed on 15 December 2025).

**Figure 6:**
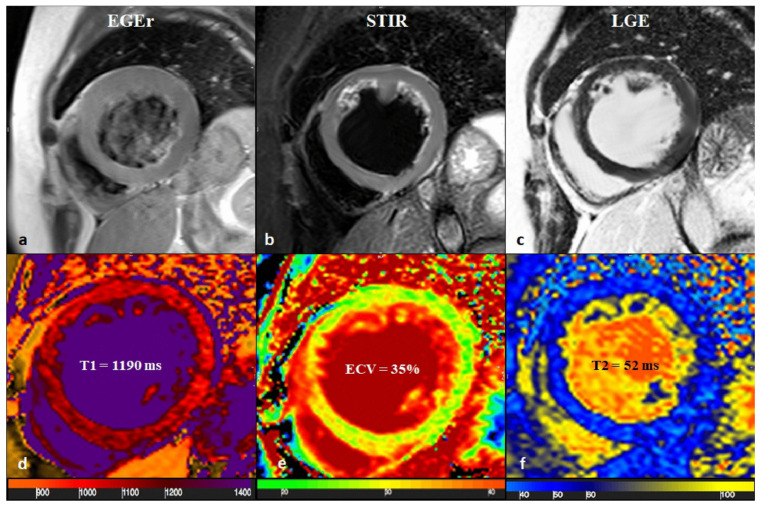
A 65-year-old female with reduced EF (24%), LV dilation (EDV/BSA: 153 ml/mq), and no significant coronary artery disease. No hyperemia (**a**, EGE ratio < 4), neither edema (**b**, T2 ratio < 2) nor LGE (**c**), was found: oLLC were negative for suspected acute myocarditis. Native T1 was severely increased (**d**), with a mid-ventricular mean value of 1190 ms; ECV (**e**) mean value was 35%, and T2 mapping was slightly increased (**f**, mean value: 52 ms). Both T1 and T2 criteria were positive according to nLLC, and the diagnosis of viral myocarditis was confirmed by EMB. EF—ejection fraction, EDV—end-diastolic volume, BSA—body surface area, EGE—early gadolinium enhancement, STIR—short tau inversion recovery, LGE—late gadolinium enhancement, oLLC—old Lake Louise Criteria, ECV—extracellular volume, nLLC—new Lake Louise Criteria, and EMB—endomyocardial biopsy. Reproduced from: Cundari, G., et al. (2021) [17]. Licensed under CC BY 4.0 (https://creativecommons.org/licenses/by/4.0/ accessed on 15 December 2025).

The inclusion of T1 and T2 mapping has improved sensitivity for detecting diffuse and global myocardial involvement, reduced reliance on contrast when contraindicated, and provided objective, quantitative measures for diagnosis and follow-up [16] (Table 2, Figure 5 and Figure 6) [17].

The removal of early gadolinium enhancement (EGE) from the mandatory diagnostic criteria in the 2018 update was a response to its documented performance variability and technical instability [9]. Theoretically, EGE identifies myocardial hyperemia and capillary leak by comparing signal intensity between the myocardium and skeletal muscle shortly after contrast injection [18]. However, while this approach aims to capture active inflammatory vasodilation, it has demonstrated inconsistent reproducibility across different imaging vendors, field strengths, and pulse sequences [9,19].

Technical limitations further undermine its clinical utility. EGE is particularly susceptible to partial volume effects at the endocardial borders and is heavily influenced by variable gadolinium kinetics, which fluctuate based on a patient’s specific cardiac output and contrast injection rate [18,20]. Clinical evaluations have highlighted high inter-observer variability, often exceeding 30% for signal ratio measurements [9]. Furthermore, while EGE may show high specificity (approx. 85–89%) in acute settings, its sensitivity significantly decreases (often below 60%) in cases of chronic inflammation or diffuse disease patterns [18,20].

In their systematic evaluation, the 2018 Lake Louise Criteria working group concluded that these limitations—compounded by a poor signal-to-noise ratio in thin myocardial walls—made EGE unsuitable for a primary diagnostic requirement [9]. Consequently, the updated guidelines relegated EGE to an optional finding, favoring the superior diagnostic accuracy and quantitative objectivity provided by T1 and T2 mapping and late gadolinium enhancement (LGE) for standardized assessment [9,21].

## 5. The Role of CMR in Determining the Prognosis of Myocarditis

CMR parameters are powerful independent predictors of outcome in myocarditis. Key prognostic indicators include the following:

- Left Ventricular Ejection Fraction (LVEF): Persistently reduced LVEF is a strong predictor of major adverse cardiac events (MACEs) and mortality [22]. Transthoracic echocardiography (TTE) serves as the initial bedside tool for LVEF assessment in acute myocarditis, reliably detecting global systolic dysfunction in fulminant cases, yet it frequently underestimates or misses regional abnormalities when LVEF remains preserved. CMR, considered the gold standard for ventricular volumetry, provides superior reproducibility and accuracy through short-axis cine stack reconstruction, particularly identifying inferolateral mid-wall dysfunction that TTE acoustic windows often obscure. In a cohort of 26 patients with CMR-confirmed acute myocarditis and preserved LVEF, TTE reported a mean LVEF of 52.7 ± 8.2%, while CMR revealed significantly lower values with patchy edema and late gadolinium enhancement (LGE) in 100% of cases, highlighting TTE’s limited sensitivity for subclinical injury [23]. These findings underscore CMR’s role as the definitive modality for LVEF evaluation and myocardial characterization in acute myocarditis, especially when TTE suggests preserved function [23,24,25].
- Late Gadolinium Enhancement—Timing and Predictive Role in Myocarditis:
  ○ The presence, extent, and pattern of LGE carry significant prognostic weight. LGE is an independent predictor of all-cause and cardiac mortality. Specifically, septal LGE and a larger extent of LGE are associated with a worse prognosis [25,26]. The evolution of LGE over time is also informative; while a reduction in LGE extent may indicate healing, persistent LGE often represents established fibrosis and a substrate for arrhythmias.
  ○ The timing of CMR re-evaluation is a decisive factor in refining this prognostic trajectory. Current longitudinal evidence suggests that follow-up imaging is most effective when performed between 6 and 12 months after the initial acute event [27,28]. This temporal window is necessary to allow for the resolution of transient inflammatory edema, ensuring that any remaining LGE accurately reflects permanent myocardial replacement fibrosis rather than acute-phase interstitial swelling. The persistence of LGE at these follow-up intervals serves as a robust marker for long-term clinical risk. For instance, in cohorts presenting with “infarct-like” myocarditis, more than 50% of patients continue to exhibit myocardial scarring at the one-year mark, a finding that correlates strongly with a higher incidence of ventricular arrhythmias [29]. Furthermore, the presence of residual LGE at follow-up is an independent predictor of adverse outcomes; meta-analytical data has demonstrated that patients with persistent enhancement face a nearly three-fold increase in the risk of MACEs, including heart failure and sudden cardiac death [27].
  ○ Consequently, serial CMR assessment is indispensable for identifying a high-risk patient phenotype that necessitates more vigilant clinical monitoring and tailored pharmacological management [27,29].
- Parametric Mapping—Timing and Predictive Role in Myocarditis:
  The prognostic utility of parametric mapping is highly dependent on the timing of the CMR exam. While baseline mapping in the acute phase is essential for diagnosis, the longitudinal evolution of these parameters provides the most critical insights into long-term cardiac risk.
  ○ Acute Phase and Follow-up Timing: The initial CMR should be performed as soon as possible after clinical presentation to capture peak T2 and T1 values, which reflect acute edema and necrosis. However, follow-up re-evaluation—typically performed 6 to 12 months later—is vital for identifying “persistent” abnormalities. At this stage, the absence of normalization in parametric values indicates a transition from acute inflammation to chronic tissue remodeling [30].
  ○ T1 Mapping and ECV (Diffuse Fibrosis): Persistently elevated native T1 and extracellular volume (ECV) values on follow-up scans are established markers of diffuse interstitial fibrosis. Research indicates that these values are significantly associated with adverse ventricular remodeling and a higher incidence of major adverse cardiovascular events (MACEs), even in patients whose left ventricular ejection fraction (LVEF) has normalized [30]. Specifically, ECV provides a quantitative measure of the expanded interstitial space, serving as a more sensitive predictor of long-term mortality and heart failure than focal LGE alone [30].
  ○ T2 Mapping (Ongoing Inflammation): Elevated T2 relaxation times are indicative of myocardial edema. From a prognostic standpoint, abnormal T2 values at the time of suspected acute myocarditis correlate strongly with poor clinical outcomes [31]. Furthermore, the failure of T2 values to normalize during follow-up suggests a state of “smoldering” or chronic inflammation, which is linked to an increased risk of disease progression and hospitalization [31].
- Myocardial Strain: Feature-tracking CMR can detect subclinical systolic dysfunction even when LVEF is preserved. Impaired global longitudinal strain (GLS) is a strong prognosticator for adverse outcomes [32]. Similarly, speckle-tracking analysis by TEE showed GLS reduced to −19.1 ± 1.8% despite normal LVEF, with regional patterns matching CMR LGE distribution in inferolateral segments [25]. Kandels et al. further demonstrated in their cohort that regional circumferential strain and LV rotation analyzed by regional layer strain exhibit a high concordance with pathological findings in cMRI [23].

In clinical practice, follow-up cardiac imaging is indicated based on the patient’s risk profile. For low-risk patients (normal LVEF, no LGE, and hemodynamic and electrical stability), in addition to an office visit, an electrocardiogram and echocardiogram are recommended at 6 months. A secondary decrease in LVEF on follow-up is uncommon but should prompt further evaluation if present.

For medium- to high-risk patients (reduced LVEF, non-sustained ventricular tachycardia (NSVT) or high-grade ventricular rhythm abnormalities, significant brady-arrythmias, LGE on CMR, increased FDG uptake on PET Scan, and hemodynamic instability or clinical heart failure) [4], a CMR is justified at 3 months in athletes considering returning to competition and can be done between 6 to 12 months for other patients. It is more sensitive to ongoing myocardial inflammation than biomarkers and echocardiography [6].

Physical exercise should be totally restricted for athletes with confirmed myocarditis for 3–6 months period considering clinical severity, duration of symptoms, LV function at onset, and extension of myocardial damage on the CMR. During this interval, only low-intensity activities of daily living are permitted [33].

**Figure 7:**
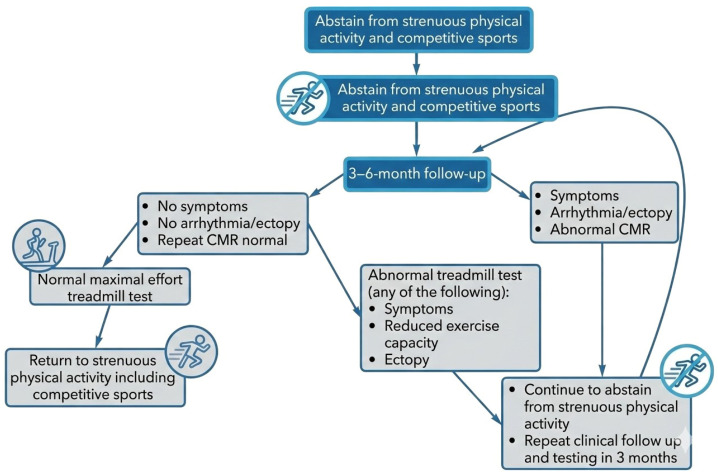
Management approach for resuming physical activity in an athlete diagnosed with acute myocarditis.

After symptom resolution, it is advisable to obtain a morphological re-evaluation, such as a CMR for medium- to high-risk patients and a transthoracic echocardiogram for low-risk patients, in addition to a 24-h ECG and an exercise stress test. Clinical clearance for athletes to resume sports participation (Class IIa, Level C) requires the fulfillment of three primary conditions: the return of left ventricular systolic function to baseline, the normalization of cardiac biomarkers, and the absence of frequent or complex arrhythmias on ambulatory ECG and exercise testing [34] (Figure 7).

In asymptomatic patients, LGE scars are considered a potential source of ventricular or supraventricular arrhythmia; therefore, regular clinical follow-up should be carried out annually. In the absence of a myocardial scar, follow-up within the first 2 years is recommended. The presence and extent of LGE alone should not preclude the athlete from resuming sports activity, but it may warrant close monitoring for ventricular hyperexcitability. In all cases, the patient must be an active participant in this decision.

## 6. CMR and False-Positive Diagnoses of Myocarditis: The Critical Role of Genetic Testing in Mimicking Conditions

A significant challenge in contemporary cardiology is distinguishing acute myocarditis from genetic cardiomyopathies that present with an inflammatory “hot phase.” These conditions can manifest with chest pain, troponin elevation, ECG changes, and ventricular dysfunction, mimicking acute myocarditis. CMR remains the primary diagnostic tool for differentiating acute myocarditis from its mimics; however, its findings can occasionally be ambiguous. It is important to note that genetic testing is not an imperative evaluation for all patients following a first episode of CMR-documented myocarditis. Instead, genetic evaluation should be considered in specific clinical scenarios where “red flags” suggest an underlying inherited cardiomyopathy, as will be discussed below [35,36,37,38].

### 6.1. Arrhythmogenic Cardiomyopathy (ACM) [39]

ACM is a genetic disorder characterized by progressive fibrofatty replacement of the myocardium, predisposing to life-threatening ventricular arrhythmias. While initially considered a disease of the right ventricle (RV), left-dominant and biventricular phenotypes are increasingly recognized [40]. During an acute inflammatory flare, often triggered by a viral infection or intense exercise, patients can present with chest pain, elevated biomarkers, and CMR evidence of myocardial edema on T2-weighted images and T2 maps, leading to an initial misdiagnosis of acute myocarditis [41].

**Figure 8:**
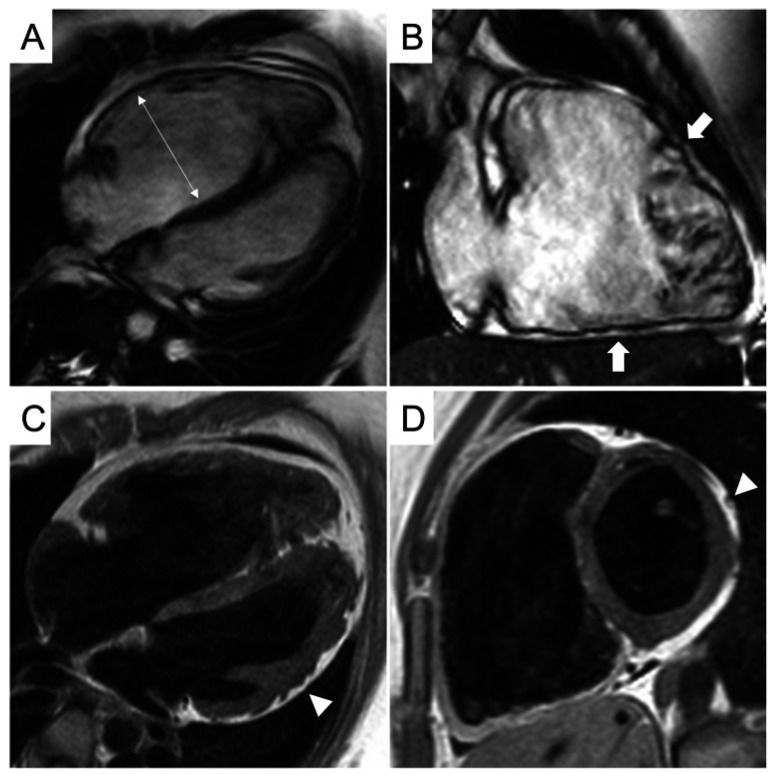
CMR frames of a patient with biventricular ACM. Morpho-functional abnormalities of the RV can be appreciated on a 4-chamber view (**A**) and right ventricular 2-chamber long-axis view (**B**) of cine images, evidencing RV dilatation (**A**, double-head arrow) and multiple sacculations of the inferior and RVOT regions (**B**, arrows). Structural alterations of the LV are showed in a 4-chamber view (**C**, arrowhead) and short-axis view (**D**, arrowhead) of T1-weighted images, where fibro-fatty infiltration of the infero-lateral LV walls becomes evident as a hyperintense signal with a typical bite-like pattern. ACM, arrhythmogenic cardiomyopathy; LV, left ventricle; RV, right ventricle; RVOT, right ventricle outflow tract. Reproduced from: Cipriani, A., et al. (2023) [39]. Licensed under CC BY 4.0 (https://creativecommons.org/licenses/by/4.0/ accessed on 15 December 2025).

CMR findings and differentiating features are as follows (Figure 8):

- LGE Pattern: The LGE in ACM is typically subepicardial or mid-myocardial, with a predilection for the basal and mid-cavity segments of the LV inferolateral wall. This pattern significantly overlaps with viral myocarditis [39].
- RV Involvement: A key differentiator is concomitant RV structural abnormality. Findings include RV dilation, systolic dysfunction (reduced RV ejection fraction), and LGE or fat infiltration in the RV free wall. However, in left-dominant ACM, RV changes can be subtle or absent.
- Regional Wall Motion Abnormalities (RWMAs): Cine imaging may show regional hypokinesia or akinesia in the LV inferolateral wall or RV, which can also be seen in myocarditis.

Given the overlap, a family history of sudden cardiac death or ACM, and the presence of characteristic ECG findings (e.g., T-wave inversions in right precordial leads or inferior/lateral leads, albeit non-specific) are crucial clues. The 2023 ESC Cardiomyopathy guidelines emphasize that in cases of suspected myocarditis with an atypical or recurrent course, the diagnosis of ACM should be considered, and genetic testing for desmosomal mutations (e.g., PKP2, DSP, and DSG2) is recommended [42]. A pathogenic variant confirms the diagnosis and enables cascade screening of family members.

### 6.2. Dilated Cardiomyopathy with an Inflammatory Trigger

A significant proportion of DCM cases have a genetic basis, with mutations in over 50 genes identified, including those encoding sarcomeric, cytoskeletal, and nuclear envelope proteins [43]. An intercurrent viral infection can act as an environmental trigger, unmasking a latent genetic predisposition by causing an inflammatory response that precipitates acute heart failure. This creates a diagnostic dilemma: is the presentation pure acute myocarditis, a genetic DCM unmasked by inflammation, or an inflammatory cardiomyopathy on a genetic background [13]?

CMR findings and differentiating features are as follows:

- LGE Pattern: While the presence of LGE favors an inflammatory or genetic etiology over a non-inflammatory cause, the pattern is not specific. Mid-wall striae of LGE in the interventricular septum are classic for non-ischemic DCM but can also be seen in chronic myocarditis. The presence of concomitant edema on T2-weighted imaging/T2 maps would favor an active inflammatory process [44].
- Ventricular Remodeling: Pronounced LV dilation and systolic dysfunction out of proportion to the extent of acute inflammatory markers might suggest an underlying chronic process like genetic DCM.

The ITAMY study and subsequent research have shown that patients with acute myocarditis who have a family history of DCM or sudden death are more likely to have a worse long-term outcome, suggesting a potential underlying genetic substrate [45]. The 2024 ACC Myocarditis Expert Consensus Decision Pathway suggests considering genetic testing in cases of DCM with a suspected inflammatory trigger, especially if there is a relevant family history or recurrent episodes [4]. Identifying a causative variant (e.g., in TTN, LMNA) shifts the management focus towards family screening and specific risk stratification for arrhythmias.

### 6.3. Lamin A/C (LMNA) Cardiomyopathy

Mutations in the LMNA gene represent a particularly malignant form of genetic DCM. Patients can present with a myocarditis-like picture, but the condition is characterized by a high incidence of progressive conduction disease and ventricular arrhythmias, often preceding significant LV dysfunction [46].

The CMR pattern is often non-specific, showing mid-wall or epicardial LGE, frequently in the basal septum or lateral wall. The key to suspicion is the clinical context: a patient presenting with “myocarditis” who also has early-onset atrial fibrillation, significant AV block, or a family history of sudden death should raise the possibility of an LMNA cardiomyopathy, prompting genetic testing [47].

### 6.4. The Imperative for Genetic Testing

The overlap between CMR phenotypes of myocarditis and genetic cardiomyopathies makes it necessary to adopt a systematic approach to genetic testing. Relying on CMR alone can lead to misdiagnosis, with profound implications for patient management and family care.

Genetic testing should be considered in the following situations:

- Recurrent “Myocarditis”: Patients with recurrent episodes of chest pain and troponin elevation, especially with preserved LV function between episodes, should be evaluated for an underlying cardiomyopathy like ACM [4,42].
- Family History: A clear family history of cardiomyopathy, sudden cardiac death, or unexplained heart failure is a strong indication for genetic testing.
- Atypical Clinical Features: The presence of prominent ventricular arrhythmias (NSVT), significant conduction disease (e.g., high-grade AV block), or disproportionate RV involvement in the setting of suspected myocarditis warrants genetic evaluation [48].
- Phenotype–Genotype Correlation: Certain CMR patterns, while not diagnostic, can guide genetic testing. For example, extensive confluent lateral wall LGE should prompt testing for desmoplakin (DSP) mutations, while septal LGE with conduction disease suggests LMNA [49].

In conclusion, CMR is the best non-invasive tool to characterize myocardial tissue in suspected myocarditis, but its limitations in distinguishing a primary inflammatory disease from a genetic cardiomyopathy with secondary inflammation must be acknowledged. A low threshold for genetic counseling and testing, guided by current international guidelines and a thorough assessment of clinical and familial data, is essential for achieving an accurate diagnosis, enabling personalized risk stratification, and ensuring appropriate family screening.

## 7. Specific Myocarditis Patterns on CMR

Beyond viral myocarditis, several systemic and toxic conditions have distinctive, though not always pathognomonic, CMR patterns that can guide the etiological diagnosis.

### 7.1. Cardiac Sarcoidosis [12,50]

Cardiac sarcoidosis (CS) is a granulomatous inflammatory disease. CMR is crucial for detecting involvement, which can be patchy and focal [51].

**Figure 9:**
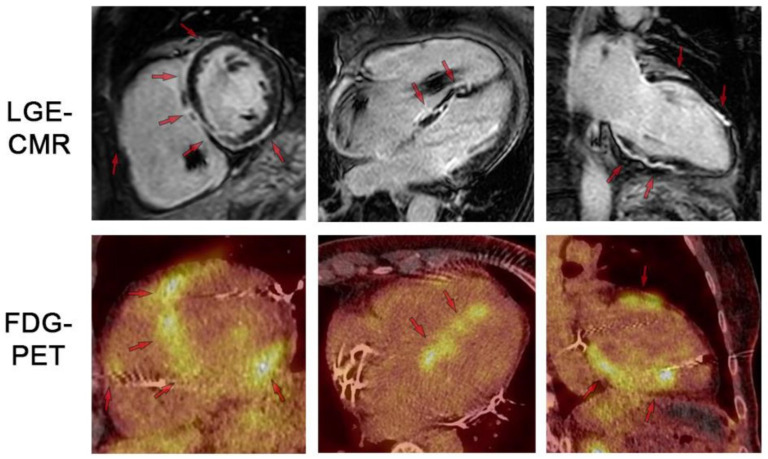
LGE-CMR and FDG-PET images for respective short-axis, 4-chamber, and 2-chamber orientations. Top row shows LGE-CMR fibrosis imaging. Arrows indicate areas of abnormal LGE in a patchy distribution pattern reflecting inflammation and/or scar. Bottom row shows fusion FDG-PET/CT showing patchy uptake in regions of scar, suggesting active inflammation. Note the artifact in the RV cavum caused by the ICD lead. LGE: late gadolinium enhancement. FDG: 18-fluordesoxyglucose positron emission tomography. Reproduced from: Korthals,. D, et al. (2024) [12]. Licensed under CC BY 4.0 (https://creativecommons.org/licenses/by/4.0/ accessed on 15 December 2025).

**Figure 10:**
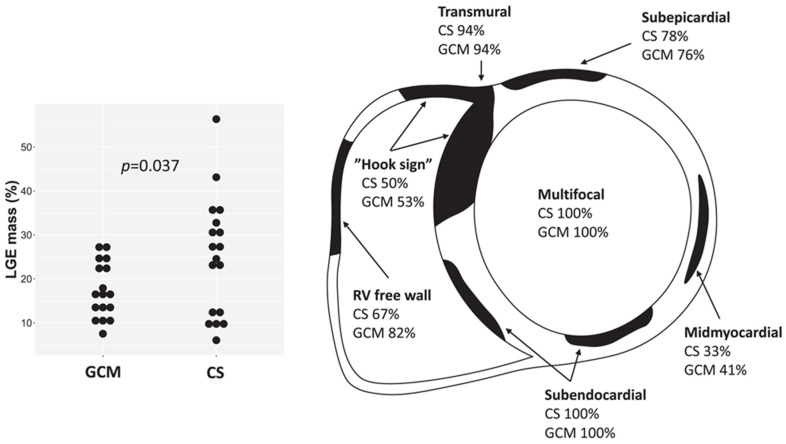
Late gadolinium enhancement (LGE) characteristics in cardiac sarcoidosis (CS) and giant cell myocarditis (GCM) on cardiac magnetic resonance, showing multifocal, transmural, and subendocardial involvement and the “hook sign.” Reproduced from: Pyhönen, P., et al (2023) [50]. Licensed under CC BY-NC 4.0 (https://creativecommons.org/licenses/by-nc/4.0/ accessed on 15 December 2025).

Characteristic CMR findings are the following (Figure 9 and Figure 10):

- LGE Pattern: The most common pattern is mid-myocardial or subepicardial LGE in the basal and mid-anteroseptal and -inferoseptal segments. A highly specific finding is the “hook sign,” where LGE extends from the septum into the right ventricular insertion point [52]. Transmural LGE can also occur, mimicking myocardial infarction.
- Mapping: Active inflammation shows elevated native T1 and T2 values in affected segments. ECV is also elevated [53].
- Functional Abnormalities: RWMAs corresponding to LGE locations, LV dilation, and reduced EF are common.
- Multimodality Imaging: 18F-FDG PET/CT is often used alongside CMR to identify active granulomatous inflammation, especially when CMR shows LGE but it is unclear if it represents active disease or fibrosis [54].

### 7.2. Immune Checkpoint Inhibitor (ICI) Myocarditis

Myocarditis is a rare but serious complication of cancer immunotherapy with ICIs. Its presentation can be fulminant, and early diagnosis is critical [55].

**Figure 11:**
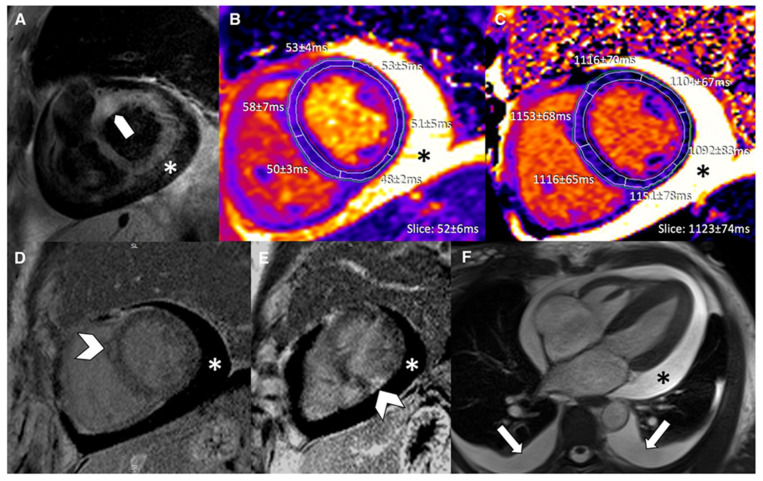
Multi-contrast cardiovascular magnetic resonance image panel in patient with immune checkpoint inhibitor myocarditis. (**A**) T2-weighted SPAIR, (**B**) T2 mapping, (**C**) native T1 mapping, (**D**,**E**) phase-sensitive inversion recovery late gadolinium enhancement imaging, and (**F**) cine steady-state free precession. Focal basal myocardial edema (block arrow), focal late gadolinium enhancement (arrowheads), moderate pericardial effusion (*), and mild bilateral pleural effusion (arrows) are demonstrated. Reproduced from: Wintersperger, B.J., et al. (2022) [58]. Licensed under CC BY-NC 4.0 (https://creativecommons.org/licenses/by-nc/4.0/ accessed on 15 December 2025).

Characteristic CMR findings are the following (Figure 11):

- Subtle Tissue Changes: A hallmark of ICI myocarditis is the frequent absence of classic LLC criteria. LGE is present in less than 50% of cases, and T2-weighted STIR images may be normal [56].
- Key Role of Mapping: Elevated native T1 mapping is the most sensitive CMR parameter for detecting ICI myocarditis, often showing global elevation even in the absence of LGE. T2 mapping may be less consistently elevated [57,58].
- Prognostic Value: The presence of septal LGE and globally elevated native T1 are independent predictors of MACEs [59].

### 7.3. Eosinophilic Myocarditis (EM)

EM is characterized by eosinophilic infiltration of the myocardium, associated with hypersensitivity, parasites, or vasculitis like EGPA [60].

**Figure 12:**
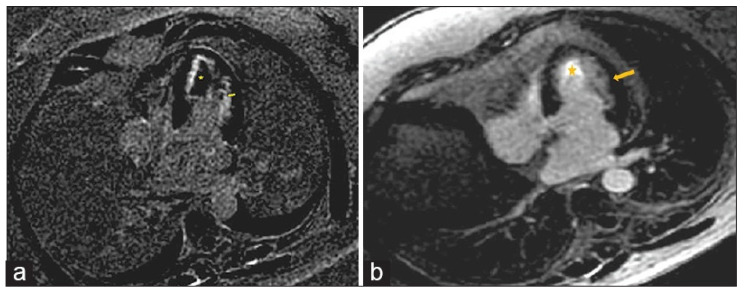
(**a**) Cardiac magnetic resonance imaging, apical four-chamber, post-contrast late gadolinium enhancement images showing extensive subendocardial fibrosis (arrow) and left ventricle mural thrombus (star). (**b**) Cardiac magnetic resonance imaging, apical four-chamber, follow-up post-contrast late gadolinium enhancement images showing significant improvement of subendocardial fibrosis (arrow) and resolution of left ventricle mural thrombus (star). Reproduced from: Taskesen, T., et al. (2022) [62]. Licensed under CC BY-NC 4.0 (https://creativecommons.org/licenses/by-nc/4.0/ accessed on 15 December 2025).

Characteristic CMR findings are the following (Figure 12):

- LGE Pattern: The most distinctive feature is subendocardial LGE, which can be extensive and circumferential, unlike the subepicardial pattern of viral myocarditis. This pattern is a hallmark of Löffler endocarditis, the chronic fibrotic stage [61].
- Associated Findings: Intramural thrombus formation is common and can be detected on cine imaging as a hypointense mass or on LGE images (which should always be reviewed alongside cine to avoid misinterpretation) [62].

### 7.4. Myocarditis Due to Human Herpes Virus 6 (HHV6) Infection

HHV6 myocarditis has a distinct, more aggressive phenotype compared to other viral causes. It presents readily as new-onset heart failure associated with malaise and conduction abnormalities like bundle branch block or even complete AV block [63,64]. Chest pain and troponin elevation are less pronounced, resulting in a longer delay to cardiac MRI [63]. HHV6 myocarditis patients have a high propensity for progression to chronic heart failure.

Characteristic CMR findings are the following:

- LGE pattern: LGE was is frequently found in the anteroseptal region, often located intramurally, without any contact with the subepicardial region [63].
- On T2 imaging, intramural hemorrhage can cause the appearance of a severe asymmetrical anteroseptal hypertrophy with typical systolic anterior motion of the mitral valve [64].
- At follow-up, it has been described that in HHV6-associated myocarditis, septal LGE persisted or even increased in some cases of combined HHV6/PVB19 myocarditis, forming a distinct mid-wall striae-like pattern within the interventricular septum [63].

## 8. Future Perspectives

Novel techniques are being developed to increase the yield of cardiac MRI in myocarditis.

One potential breakthrough in research is the development of molecular imaging. For instance, a study in a murine model used F-fluorine CMR in vivo to detect immune cell infiltration in autoimmune myocarditis. Perfluorocarbons are injected intravenously and then taken up by monocytes/macrophages, which are specifically recruited to areas of inflamed myocardium [65]. By labeling macrophages and monocytes, these techniques can pinpoint active cellular infiltration, providing a more specific biological diagnosis than the generalized tissue responses (edema/hyperemia) currently detected by the Lake Louise Criteria.

Artificial intelligence and machine learning are increasingly being applied in CMR imaging to enhance the diagnosis of acute myocarditis. AI-based approaches have shown potential to match human diagnostic performance and support phenotyping while also saving clinicians’ time and aiding decision making by providing more robust analytical tools. Applications in CMR include automatic segmentation of myocardial scars and edema, improved classification and detection of myocarditis features, and the use of deep learning and explainable AI to interpret CMR images and assist early diagnosis. However, most studies to date are limited by small, single-center cohorts and a lack of relevant cardiac disease controls, underscoring the need for larger, well-characterized datasets and further validation to facilitate clinical integration of AI in myocarditis diagnostics [66].

New perspectives in CMR for myocarditis include advances that enhance patient comfort and diagnostic precision through improved image acquisition and processing. Techniques such as high-resolution 3D LGE enable the detection of smaller and more focal areas of myocardial injury. Free breathing and motion correction methods improve image quality, especially for patients unable to hold their breath consistently. Additionally, cine imaging innovations, including black blood cine and magnetic resonance fingerprinting cine, allow for simultaneous assessment of myocardial tissue characteristics and wall motion. Deep learning applied to cine imaging and perfusion mapping accelerates image acquisition and reconstruction while enhancing diagnostic accuracy. Exercise stress CMR is emerging with new compatible equipment and image quality improvements, broadening clinical applications including myocarditis evaluation.

Among cutting-edge methods are diffusion tensor imaging (DTI) and 4D flow [67], which offer novel insights into myocardial microstructure and intracardiac hemodynamics. DTI non-invasively characterizes the 3D myocardial fiber orientation and microstructural integrity by measuring the diffusion of water molecules constrained by tissue barriers, without requiring contrast agents. It provides dynamic information through multiple cardiac phases, improving the understanding of myocardial laminar structure and its interaction with cardiac mechanics. On the other hand, 4D flow imaging captures time-resolved three-dimensional blood flow velocity data, enabling comprehensive evaluation of flow patterns and shear stresses within the heart and great vessels. This facilitates assessment of diastolic function, valvular lesions, and other flow-related abnormalities difficult to characterize by other modalities. Together, these techniques augment the capability of CMR to non-invasively phenotype myocardial disease and support personalized diagnosis and management in myocarditis.

Strain analysis in cardiac MRI provides sensitive markers for myocardial deformation abnormalities that can be present even when traditional measures like left ventricular ejection fraction are preserved. Feature-tracking strain on CMR has demonstrated high diagnostic sensitivity for myocarditis and offers incremental prognostic value beyond ejection fraction and LGE, associating with adverse clinical outcomes [68].

Finally, the clinical utility of CMR in acute myocarditis is often hindered by patient tachycardia, which can cause motion artifacts and lower image quality. Several technical advances are poised to diminish the importance of heart rate in the diagnostic evaluation like Compressed Sensing technology, Deep Learning (DL)-Based Reconstruction, and pulsatile motion-based sensors that significantly reduce acquisition time and reliance on good ECG signal quality [69,70,71].

## Figures and Tables

**Table 1:** Etiological classification of myocarditis. This table summarizes the diverse etiological landscape of myocarditis, categorized by the nature of the triggering insult. It encompasses traditional infectious agents, including the most prevalent DNA and RNA viruses, as well as emerging immune-mediated triggers such as mRNA vaccines and immune checkpoint inhibitors (ICIs). Furthermore, the table includes genetic cardiomyopathies that frequently present with an inflammatory “hot phase” (genetic mimics), which are critical for differential diagnosis in clinical practice. Approximately 50% of clinical cases remain idiopathic despite extensive diagnostic evaluation. ACM: Arrhythmogenic Cardiomyopathy; CMV: Cytomegalovirus; EBV: Epstein–Barr Virus; FLNC: Filamin C; HHV-6: Human Herpesvirus 6; HIV: Human Immunodeficiency Virus; HSV: Herpes Simplex Virus; ICIs: Immune Checkpoint Inhibitors; LMNA: Lamin A/C; mRNA: Messenger Ribonucleic Acid; PKP2: Plakophilin-2; DSP: Desmoplakin; SLE: Systemic Lupus Erythematosus.

Category	Sub-Category	Examples/Specific Triggers
Infectious Agents	Viral (DNA)	Parvovirus 819, Adenovirus, HHV-6, EBV, CMV, HSV
	Viral (RNA)	Coxsackievirus (A and B), SARS-CoV-2, Influenza A/B, HIV, Hepatitis C
	Bacterial	*Staphylococcus, Streptococcus, Corynebacterium diphtheriae*, Mycoplasma
	Spirochetes	*Borrelia burgdorferi* (Lyme disease), Leptospira
	Fungal/Protozoal	Aspergillus, Candida/*Trypanosoma cruzi* (Chagas), *Toxoplasma gondii*
2. Immune Mediated	Autoimmune Disorders	SLE, Sarcoidosis, Giant cell myocarditis, Rheumatoid arthritis
	Hypersensitivity	Allergic reactions to Penicillin, Clozapine, Sulfonamides, Cephalosporins
	Vaccine related	mRNA COVID-19 vaccines, Smallpox vaccine
3. Toxic & Drugs	Oncology Therapies	Immune Checkpoint Inhibitors (ICls), Anthracyclines, Trastuzumab
	Recreational/Toxins	Cocaine, Amphetamines, Alcohol, Carbon monoxide
	Heavy Metals	Iron (Hemochromatosis), Copper, Lead
	Venoms	Scorpion stings, Snake bites, Bee/Wasp stings
4. Physical Agents		Radiation therapy, Electric shock, Heatstroke
5. Genetic Mimics	Desmosomal/Others	ACM (e.g., PKP2, DSP), LMNA, FLNC mutations
6. Idiopathic		No specific cause identified (approx. 50% of cases)

**Table 2:** Comparison of the 2009 and 2018 Updated Lake Louise Criteria (LLC) for myocarditis. LGE: late gadolinium enhancement; EGE: early gadolinium enhancement.

Feature	2009 Original LLC	2018 Updated LLC
T1-Based Criteria (Injury/Fibrosis)	LGE: Focal non-ischemic lesion (subepicardial/mid-myocardial)	Any of the following: Elevated native T1relaxation times Presence of non-ischemic LGE Increased Extracellular Volume (ECV)
T2-Based Criteria (Edema)	T2-weighted images: Regional or global edema (T2 signal intensity ratio 2.0)	Any of the following: Elevated native T2 relaxation times Regional or global increase in T2 signal intensity on T2-weighted images
Vascular Criteria (Hyperemia)	EGE: Global myocardial hyperemia and capillary leak	Removed from mandatory criteria due to variable performance and subjectivity
Primary Advantages	Established first standardized CMR approach for myocarditis	Improved sensitivity for diffuse/global disease; provides objective, quantitative measures; reduces reliance on contrast
Limitations	Subjective imaging; susceptible to artifacts; low sensitivity in chronic or diffuse cases	Requires advanced mapping software and established local reference ranges for T1/T2 values

## Data Availability

No new data were created or analyzed in this study.
